# Licit prescription drug use in a Swedish population according to age, gender and socioeconomic status after adjusting for level of multi-morbidity

**DOI:** 10.1186/1471-2458-12-575

**Published:** 2012-07-31

**Authors:** Kristine Thorell, Jessica Skoog, Andrzej Zielinski, Lars Borgquist, Anders Halling

**Affiliations:** 1 Blekinge Centre of Competence, SE-371 81, Karlskrona, Sweden; 2 Department of Clinical Sciences, Malmö, General Practice/Family Medicine, Lund University, SE-205 02, Malmö, Sweden; 3 Department of Clinical Sciences, Malmö, Center for Primary Health Care Research, Lund University, Region Skåne, SE-205 02, Malmö, Sweden; 4 Department of Medical and Health Sciences, General Practice, Linköping University, SE-581 83, Linköping, Sweden; 5 Research Unit for General Practice, Institute of Public Health, University of Southern Denmark, J.B. Winsløws Vej 9a, DK-5000, Odense C, Denmark

**Keywords:** Age, Gender, Multi-morbidity, Licit prescription drug use, Case-mix, Multi-morbidity, Primary health care, Sweden

## Abstract

**Background:**

There is a great variability in licit prescription drug use in the population and among patients. Factors other than purely medical ones have proven to be of importance for the prescribing of licit drugs. For example, individuals with a high age, female gender and low socioeconomic status are more likely to use licit prescription drugs. However, these results have not been adjusted for multi-morbidity level. In this study we investigate the odds of using licit prescription drugs among individuals in the population and the rate of licit prescription drug use among patients depending on gender, age and socioeconomic status after adjustment for multi-morbidity level.

**Methods:**

The study was carried out on the total population aged 20 years or older in Östergötland county with about 400 000 inhabitants in year 2006. The Johns Hopkins ACG Case-mix was used as a proxy for the individual level of multi-morbidity in the population to which we have related the odds ratio for individuals and incidence rate ratio (IRR) for patients of using licit prescription drugs, defined daily doses (DDDs) and total costs of licit prescription drugs after adjusting for age, gender and socioeconomic factors (educational and income level).

**Results:**

After adjustment for multi-morbidity level male individuals had less than half the odds of using licit prescription drugs (OR 0.41 (95% CI 0.40-0.42)) compared to female individuals. Among the patients, males had higher total costs (IRR 1.14 (95% CI 1.13-1.15)). Individuals above 80 years had nine times the odds of using licit prescription drugs (OR 9.09 (95% CI 8.33-10.00)) despite adjustment for multi-morbidity. Patients in the highest education and income level had the lowest DDDs (IRR 0.78 (95% CI 0.76-0.80), IRR 0.73 (95% CI 0.71-0.74)) after adjustment for multi-morbidity level.

**Conclusions:**

This paper shows that there is a great variability in licit prescription drug use associated with gender, age and socioeconomic status, which is not dependent on level of multi-morbidity.

## Background

Prescription of licit drugs is one of the most frequently used methods to prevent and treat diseases in health care [1]. Due to the high costs and potential risks associated with inappropriate licit prescription drug utilization it is of importance that drugs are prescribed in a correct and responsible way [2]. The Swedish population of elderly is increasing [3] and the prevalence of chronic disease increases with age, thus also increasing the number of elderly patients with one or more licit prescription drugs [2]. The licit prescription drug costs in Sweden have escalated during the past years [4]. Due to new and improved licit prescription drugs more medical conditions can be treated. New licit prescription drugs are developed and continuously released onto the market and older ones get new indications. This contributes to the increase in licit prescription drug use and costs for licit prescription drugs. It also increases the risk of polypharmacy (defined as the use of five or more daily prescribed drugs), which can cause interactions, adverse drug reactions and lead to an increased number of hospital admissions [5-7]. In 2007 the licit prescription drug costs amounted to SEK 32.8 billion (1 Swedish crown (SEK) ≈ 0.1 Euro) [4]. Factors other than purely medical ones are of importance for licit prescription drug use. Women are more likely to use licit prescription drugs and the total costs of licit prescription drug use in women are higher than in men [2,8-10], while men have a higher cost per dose [1,11,12]. Individuals from lower social class have a higher usage of licit prescription drugs, but at the same time the cost per dose is in comparison the lowest [11-13].

The prevalence of chronic illness increases with age, and therefore age increases the risk of receiving licit prescription drug treatment. This is illustrated by the fact that the elderly (≥65 years) in Sweden constituted 18% of the total population during 2007 [14], but they accounted for 41% of the total licit prescription drug costs [15]. Our aim was to investigate the odds of using licit prescription drugs among individuals in the population and the rate of licit prescription drug use among patients depending on age, gender and socioeconomic status after adjusting for multi-morbidity level. Only a few studies have taken the individual patient’s multi-morbidity level into account when studying what influences licit prescription drug use [8,13]. Neither one of these studies have taken all the patient’s diagnoses into account when calculating the level of multi-morbidity. We used the Johns Hopkins ACG Case-Mix System to calculate the level of multi-morbidity. The ACG Case-Mix system is based on a theory that multi-morbidity corresponds to a certain need of health care resources. The multi-morbidity is measured by analysing all diagnoses recorded during a time period using an ACG Case-Mix algorithm. This algorithm assigns each individual with an ACG index. This index is categorized into six Resource Utilization Bands (RUB) or multi-morbidity levels [16,17].

## Methods

### Study population

The study was carried out during 2006 on the total population aged 20 years or older in Östergötland county with about 400 000 inhabitants. Östergötland county is situated about 200 km southwest of Stockholm. The age demography in Östergötland is similar to that of the rest of Sweden [18]. Data on the population’s age, gender, diagnoses in primary health care (PHC) and secondary care were obtained through the Care Data Warehouse in Östergötland (CDWÖ). Information on this register has been described earlier [19]. The data in this register contained information from both private and public caregivers. The study was approved by the research ethics committee at Linköping University (Dnr 147/05 and 29/06).

### Dependent variables

Information on the use of licit prescription drugs on the individual level was obtained through the Swedish Prescribed Drug Register at the National Board of Health and Welfare, which collects information from the National Corporation of Swedish Pharmacies (Apoteket AB), which at the time of the study had a monopoly on sales of licit prescribed drugs. All the licit prescribed drugs were tracked through Apoteket AB for the time being. Two dependent variables were used in this study: 1. Use of licit prescription drugs in Defined Daily Doses (DDDs) during 2006. 2. Total costs of licit prescription drug use (Apoteket AB sales price to customers (AUP)) during 2006 in SEKs (1 Swedish crown (SEK) ≈ 0.1 Euro). DDDs are defined by WHO as a statistical measure of licit prescribed drug consumption and is not to be confused with the number of doses prescribed by the physicians. DDDs are used to standardize the comparison of licit prescription drug use between different licit prescription drugs [20]. In this study we have used the collected DDDs for each patient. Over the counter drugs are not included in this study. AUP is the sales price used by the pharmacies when selling licit prescription drugs. It is determined by the Dental and Pharmaceutical Benefits Agency [21].

### Independent variables

#### Multi-morbidity

Multi-morbidity was calculated using the Johns Hopkins ACG Case-Mix System. The ACG Case-Mix system is based on the theory that multi-morbidity corresponds to a certain need of health care resources. The multi-morbidity is measured as a sum of diagnoses recorded during a defined period of time. The ACG Case-Mix system has previously been described [16,17,22,23]. Individuals without need of health care according to the ACG Case-Mix system are placed in Resource Utilization Band 0 (RUB 0) and individuals with a very high degree of need for health care resources are placed in RUB 5. For example, preventive interventions correspond to RUB 1, a single chronic diagnosis could correspond to RUB 3 and a certain combination of chronic diagnoses corresponds to RUB 4 or RUB 5. All diagnoses from primary and secondary care from 2006 were obtained through the Care Data Warehouse in Östergötland (CDWÖ) [19].

#### Socioeconomics

There are many different variables to describe socioeconomics, e.g. income, education, environment and occupation [24-26]. We used income and education, two commonly used variables to describe socioeconomics, and they have been shown not to be interchangeable [26]. The education variable was divided into four levels: 1. Primary school not completed (<9 years). 2. Primary school completed (9–10 years) 3. Secondary school (10- 12 years) 4. Higher education (>12 years). The disposable income was divided into quartiles from the highest to the lowest with equal number of individuals in each quartile. For a professional career the number of years in school is not as important as having a formal degree. Consequently, whether or not an individual has completed primary school has great impact on professional life and possible social position, even if there is only one school year’s difference [26]. The information on individual level of education and income was obtained from Statistics Sweden [27]. Due to the fact that young people under 20 years of age had no complete record of education and no or low income, this group was excluded from the study. The information on educational level was also to a high degree missing in people 70 years or older and therefore this group was excluded when the effect of educational level was analysed.

### Statistical analysis

In this study we used STATA version 10 (Stata Corporation, Texas, USA) for statistical analyses. A descriptive analysis was performed on the use of licit prescription drugs in the population. The categorical variables were compared using chi-square test. Due to the high number in the population not using licit prescription drugs Poisson regression analysis was not considered valid. The best statistical analysis that could model our data was considered to be zero inflated negative binomial regression, which performs two simultaneous analyses [28]. One analysis is similar to logistic regression and answers the question on what the odds are of belonging to the individuals in the population without licit prescription drug use and also gives an odds ratio (OR). These odds ratios were then inverted to show odds ratio of using licit prescription drugs. The other analysis is similar to Poisson regression and answers the question of what the effect is of increasing the independent variable by one unit among patients (those that use licit prescription drugs) and gives an incidence rate ratio (IRR). This provided two different analyses of licit prescription drug use for individuals in the population and two analyses for patients in the population. The dichotomous models for DDDs and costs for individuals gave similar results, so we used only the one for DDDs. We generated three different models for each analysis: Model 1 adjusted for multi-morbidity, Model 2 adjusted for multi-morbidity and gender, Model 3 adjusted for multi-morbidity, gender and age.

## Results

### Study population

A majority (66%) of the study population used licit prescription drugs during 2006 and a significantly higher proportion of females used licit prescription drugs (Table 1). In the age group 70 years or older and in RUB 2 or higher 80% or more used licit prescription drugs. Among patients using licit prescription drugs 60% were 50 years or older. A lower proportion of those with higher SES used licit prescription drugs (Table 1).

**Table 1:** Characteristics of the population’s licit prescription drug use

**Licit prescription drug use in total population**			
**Variables**		**Yes**	**No**
		N(%)	N(%)
All		205827 (66)	108150 (35)
Gender	Female	121682 (77)	37021 (23)
	Male	84145 (54)	71129 (46)
Age	20-29	23916 (51)	23289 (49)
	30-39	27666 (53)	24568 (47)
	40-49	30419 (56)	24293 (44)
	50-59	34946 (65)	19045 (35)
	60-69	36745 (73)	11376 (34)
	70-79	27643 (87)	4038 (13)
	80-	24492 (94)	1541 (6)
Multi-morbidity			
level	0	26822 (26)	75013 (74)
	1	30364 (69)	13491 (31)
	2	51674 (80)	12913 (20)
	3	82988 (93)	6595 (7)
	4	10775 (99)	126 (1)
	5	3204 (99.6)	12 (0.4)
Educational level*	1	15377 (73)	5732 (27)
up to 60–69years	2	16292 (62)	10003 (38)
	3	74886 (60)	50695 (40)
	4	45296 (56)	35001 (44)
Income level**	1	55260 (70)	23185 (30)
	2	59030 (75)	19415 (25)
	3	48816 (62)	29630 (38)
	4	42720 (55)	35724 (45)

*Missing 2981, **Missing 197.

N– Number of observations.

### Gender

After adjustment of multi-morbidity male individuals had half the odds of using licit prescription drugs compared to females (Table 2 OR 0.41 (95% CI 0.40- 0.42)). However, male patients had decreased rates of DDDs (IRR 0.97 (95% CI 0.96- 0.98)) and increased rates of costs (IRR 1.14 (95% CI 1.13-1.15)) compared to female patients (Table 3).

**Table 2:** Odds ratio of licit prescription drug use in the population

**Variables**		**OR (95%)**	**P-value**
Gender^1^			
	Female	1	
	Male	0.41 (0.40-0.42)	0.000
Age (years)^2^			
	20-29	1	
	30-39	1.04 (1.001.08)	0.027
	40-49	1.26 (1.221.08)	0.000
	50-59	1.85 (1.781.88)	0.000
	60-69	3.03 (2.943.03)	0.000
	70-79	4.76 (4.545.00)	0.000
	80	9.09 (8.3310.00)	0.000
Educational level3 up to 60–69 years			
	1	1	
	2	0.97 (0.92-1.02)*	0.261
	3	0.94 (0.90-0.99)	0.014
	4	0.89 (0.85-0.93)	0.000
Income level^3^			
		1	
	2	1.16 (1.14-1.20)	0.000
	3	1.06 (1.03-1.09)	0.000
	4	1.05 (1.02-1.08)	0.001

^1^Adjusted for multi-morbidity, ^2^Adjusted for multi-morbidity and gender, ^3^Adjusted for multimorbidity, gender and age.

OR – odds ratio, 95% – confidence interval, DDDs – Defined Daily Doses.

**Table 3:** The incidence rate ratio of using licit prescription drugs and costs for licit prescription drug use among patients

**Variables**		**DDDs**		**Costs**	
		**IRR (95%)**	**P-value**	**IRR (95%)**	**P-value**
Gender^1^					
	Female	1		1	
	Male	0.97 (0.96–0.98)	0.000	1.14 (1.13–1.15)	0.000
Age (years)^2^					
	20–29	1		1	
	30–39	1.07 (1.05–1.09)	0.000	1.07 (1.05–1.10)	0.000
	40–49	1.50 (1.47–1.53)	0.000	1.42 (1.39–1.46)	0.000
	50–59	2.18 (2.14–2.22)	0.000	1.63 (1.59–1.67)	0.000
	60–69	2.97 (2.91–3.03)	0.000	1.88 (1.84–1.92)	0.000
	70–79	3.68 (3.60–3.76)	0.000	1.95 (1.90–1.99)	0.000
	80	4.50 (4.40–4.60)	0.000	1.95 (1.91–2.00)	0.000
Educational level^3^					
up to 60–69 years	1	1		1	
	2	0.99 (0.96–1.02)*	0.341	1.05 (1.02–1.09)	0.002
	3	0.85 (0.83–0.87)	0.000	0.96 (0.93–0.98)	0.002
	4	0.78 (0.76–0.80)	0.000	0.92 (0.89–0.94)	0.000
Income level^3^					
	1	1		1	
	2	1.00 (0.99–1.02)*	0.657	0.97 (0.96–0.99)	0.001
	3	0.81 (0.80–0.82)	0.000	0.83 (0.82–0.85)	0.000
	4	0.73 (0.71–0.74)	0.000	0.71 (0.70–0.72)	0.000

IRR – incidence rate ratio, 95% – confidence interval, DDDs – defined daily doses

^1^Adjusted for multi-morbidity, ^2^Adjusted for multi-morbidity and gender, ^3^Adjusted for multimorbidity,

gender and age.

### Age

Age increased the odds of using licit prescription drugs and the oldest individuals in the population (>80 years) had the highest odds of licit prescription drug use (Table 2). Age increased the rate of both DDDs and costs for patients (Table 3).

### Socioeconomics

The highest educational level in individuals decreased the odds of licit prescription drug use by 11%. For individuals the lowest income level had the lowest odds of licit prescription drug use and the second lowest level of income had the highest odds of licit prescription drug use, increased by 16% (Table 2). The highest educational level of patients was associated with a reduced rate of DDDs of 21% and a reduced rate of costs of 8% (Table 3). The highest income level among patients reduced the rate of DDDs by 27% and the rate of costs by 29% (Table 3).

## Discussion

### Gender

The finding that females have higher odds of licit prescription drug use than males in the population although males represent higher costs has previously been shown [2,29]. What we have shown here is that the difference between female and male individuals in the population and among patients remains after adjustment for multi-morbidity. One hypothesis is that males and females have different healthcare seeking behaviour. Statistics from USA indicate that females seek more preventive healthcare than males, but emergency treatment at the same rate [30]. This could indicate that men seek health care later than females and could partly explain why males have lower odds of licit prescription drug use despite the same multi-morbidity [31]. However, research in this area has not found any clear evidence to support that hypothesis. It has also been discussed that females and males have different ways of describing symptoms and therefore have different odds of licit prescription drug use [32]. The differences in prevalence of disease between the genders have been discussed before [8]. For example, males have more licit prescription drug treatment for cardiovascular disease while females have more licit prescription drug treatment for anxiety and depression diagnoses [29]. How this difference in diagnosis prevalence affects licit prescription drug use and costs is not examined here but we cannot exclude the possibility that it may affect our results. Even though it has not been examined in this study we cannot exclude that the licit prescription drugs are being prescribed in a biased way related to doctors’ characteristics.

### Age

Despite the same multi-morbidity level age increased the odds of licit prescription drug use in individuals and rate of use in patients. Among elderly one can talk about the “prescribing cascade”. It is described as starting as an adverse drug reaction that is misinterpreted as a new medical condition. A new drug is then prescribed, and the patient is put at risk of developing additional adverse effects relating to this potentially unnecessary treatment [1].

Another hypothesis as to why elderly, despite same multi-morbidity, have higher odds of licit prescription drug use is that the evaluation of the licit prescription drug treatment may have been neglected.

Different treatment ways for different age groups can affect the odds of licit prescription drug use in the population and rate of use in patients. Younger individuals are probably more likely to be recommended lifestyle changes as a first point of action before licit prescription drug treatment to treat for example hypertension. The duration of a disease in a patient also influences the DDDs for the same disease. Hypertension may again be used as a good example since physiological changes increases the blood pressure the older the individuals get. It is worrying that elderly, who are the most sensitive to drugs, have the highest use of licit prescription drugs. All clinicians need to get better at reviewing licit prescription drug treatment in the elderly and phase out treatment that no longer has an indication.

### Socioeconomics

After adjustment for multi-morbidity level, gender and age individuals in the lowest income level had the lowest odds of licit prescription drug use in the population, while the second lowest income level had the highest odds of licit prescription drug use. We had expected the lowest income level to have the highest odds of licit prescription drug use. Our interpretation to the result is that it could depend on poor economy in the lowest income level, preventing these individuals from collecting their licit prescription drugs. This is interesting since Sweden has a high cost threshold system for licit prescription drugs [21]. Provided that the licit prescription drug is granted reimbursement status, the individual does not pay more than SEK 1800 (~180 EUR) per 12-month period for licit prescription drugs. The finding that the lowest income level has the lowest odds of licit prescription drug use may indicate that there are individuals in the population who despite a pharmaceutical benefit system do not collect their prescribed drugs. This theory is supported by the fact that there is no statistical significant difference in the odds of licit prescription drug use between the lowest and second lowest level of education (Table 2).

Former studies have shown differences in healthcare seeking behaviour with higher consultation rates among individuals in lower social classes [33]. Even though it is not studied here, it is possible that higher rates of healthcare seeking increase the licit drug prescribing. Individuals with low education have proved to a lesser extent to act on information concerning health risks such as smoking [34]. If this fact also affects the capacity to act on doctors’ recommendations of lifestyle changes it may lead to increased licit drug prescribing.

### Limitations

Our study includes the total population of Östergötland county, which is representative of the rest of Sweden [27].

Since individuals 70 years or older was excluded when analysing educational level it was impossible to adjust for both income and education consecutively and therefore we analysed these variables separately. The missing data in the rest of the variables were minor and did not affect the results.

ACG Case-Mix uses diagnoses to calculate multi-morbidity and is therefore dependent on the quality of registration of diagnoses [16]. The recording of diagnoses in this study is not validated. However, a prior study in Sweden has shown that 75% of the population had at least one diagnosis-registered encounter at a GP during a three-year period [35]. During 2006 Östergötland did not use ACG Case-Mix for reimbursement and therefore the risk of up coding is unlikely. It is more likely that there is an under-registration [36].We were not able to assess illicit drug use and these drugs are not included in this study. Data from Medical Products Agency indicates that 11% of the Swedish population has bought prescription drugs from non-approved pharmacies during 2011[37].

In this study we have data on licit prescription drugs that were collected from the pharmacies but we do not have information on to what extent the patients actually used them.

### Future studies

This study has provided an important insight into which variables influence licit prescription drug use. Multi-morbidity is clearly not the only factor affecting licit prescription drug use. There are evident differences between gender, age and socioeconomic status. Future studies should focus on what these differences depend on.

## Conclusion

This study shows that there is a great variability in licit prescription drug use with regard to gender, age and socioeconomic status that cannot be explained by different levels of multi-morbidity. On the contrary, the studied factors - gender, age and socioeconomic status - seem to have a high impact on licit prescription drug use. This clearly illustrates that there are more variables than multi-morbidity influencing a patient’s licit prescription drug use. It is of utmost importance that politicians, officials and doctors are aware of that fact.

## Competing interests

The author(s) declare that they have no competing interests.

## Authors’ contributions

KT and JBS drafted the manuscript and participated in the design of the study. AZ helped draft the manuscript. LB helped draft the manuscript. AH preformed the statistical analysis and drafting of the manuscript, handled the data set and designed the study. All authors read and approved the final manuscript.

## Key points of the article

Age, gender and socioeconomic status are important factors for prescription drug treatment in a population, even after adjusting for multi-morbidity.

## Pre-publication history

The pre-publication history for this paper can be accessed here:

http://www.biomedcentral.com/1471-2458/12/575/prepub

## References

[B1] Rochon PA Gurwitz JH Optimising drug treatment for elderly people: the prescribing cascade BMJ 1997 315 7115 1096 1099 10.1136/bmj.315.7115.1096 9366745 PMC2127690

[B2] Correa-de-Araujo R Miller GE Banthin JS Trinh Y Gender differences in drug use and expenditures in a privately insured population of older adults J Womens Health (Larchmt) 2005 14 1 73 81 10.1089/jwh.2005.14.73 15692281

[B3] Ekonomifakta, http://www.ekonomifakta.se/sv/fakta/arbetsmarknad/befolkning/befolkningsstruktu

[B4] Apotekensservice AB, http://www.apotekensservice.se/Om-Apotekens-Service/Uppdrag/Statistik/

[B5] Haider SI Johnell K Thorslund M Fastbom J Analysis of the association between polypharmacy and socioeconomic position among elderly aged>or =77years in Sweden Clin Ther 2008 30 2 419 427 10.1016/j.clinthera.2008.02.010 18343279

[B6] Holland R Lenaghan E Harvey I Smith R Shepstone L Lipp A Christou M Evans D Hand C Does home based medication review keep older people out of hospital? The HOMER randomised controlled trial Br Med J 2005 330 7486 293 295 10.1136/bmj.38338.674583.AE 15665005 PMC548182

[B7] Fastbom J Ökat äkemedelsintag bland äldre innebär ökad risk för problem Läkartidningen 2001 98 14 1674 1679 11379169

[B8] Stock SA Stollenwerk B Redaelli M Civello D Lauterbach KW Sex differences in treatment patterns of six chronic diseases: an analysis from the German statutory health insurance J Womens Health (Larchmt) 2008 17 3 343 354 10.1089/jwh.2007.0422 18338965

[B9] Roe CM McNamara AM Motheral BR Use of chronic medications among a large, commercially-insured US population Pharmacoepidemiol Drug Saf 2002 11 4 301 309 10.1002/pds.700 12138598

[B10] Fernandez-Liz E Modamio P Catalan A Lastra CF Rodriguez T Marino EL Identifying how age and gender influence prescription drug use in a primary health care environment in Catalonia, Spain Br J Clin Pharmacol 2008 65 3 407 417 10.1111/j.1365-2125.2007.03029.x 17922886 PMC2291251

[B11] Engstrom S Magnusson H Enthoven P Walter L Thorell K Halling A Borgquist L Social status affects drug costs and health care costs. A registry study shows that Care Choice should take socioeconomic factors into account Lakartidningen 2009 106 48 3248 3250 3252–3 20101836

[B12] Chen YF Dewey ME Avery AJ Analysis Group of The MRCCFA Study. The Medical Research Council Cognitive Function and Ageing Study (MRC CFAS): Self-reported medication use for older people in England and Wales J Clin Pharm Ther 2001 26 2 129 140 10.1046/j.1365-2710.2001.00333.x 11350536

[B13] Haider SI Johnell K Weitoft GR Thorslund M Fastbom J The influence of educational level on polypharmacy and inappropriate drug use: a register-based study of more than 600,000 older people J Am Geriatr Soc 2009 57 1 62 69 10.1111/j.1532-5415.2008.02040.x 19054196

[B14] Tabeller över Sveriges befolkning 2007 Korrigerad version 2010 02 18 http://www.scb.se/Pages/PublishingCalendarViewInfo 259923.aspx?PublObjId=6636

[B15] Läkemedel- statistikförår 2007 2008 2008 46 1 23 http://www.socialstyrelsen.se/publikationer2008/2008-46-1

[B16] Starfield B Weiner J Mumford L Steinwachs D Ambulatory care groups: a categorization of diagnoses for research and management Health Serv Res 1991 26 1 53 74 1901841 PMC1069810

[B17] Zielinski A Kronogard M Lenhoff H Halling A Validation of ACG Case-mix for equitable resource allocation in Swedish primary health care BMC Public Health 2009 9 347 10.1186/1471-2458-9-347 19765286 PMC2755480

[B18] Animerade befolkningspyramider 1968–2010 - från SCBhttp://www.h.scb.se/kommunfakta/pyramider/index.asp1968

[B19] Wirehn AB Ostgren CJ Carstensen JM Age and gender differences in the impact of diabetes on the prevalence of ischemic heart disease: a population-based register study Diabetes Res Clin Pract 2008 79 3 497 502 10.1016/j.diabres.2007.10.009 18006174

[B20] World Health Organization, http://www.whocc.no/ddd/definition_and_general_considera/

[B21] The Dental and Pharmaceutical Benefits Agency, http://www.tlv.se/in-english- old/medicines-new/the-swedish-high-cost-threshold/

[B22] Weiner JP Starfield BH Steinwachs DM Mumford LM Development and application of a population-oriented measure of ambulatory care case-mix Med Care 1991 29 5 452 472 10.1097/00005650-199105000-00006 1902278

[B23] Orueta JF Lopez-De-Munain J Baez K Aiarzaguena JM Aranguren JI Pedrero E Application of the ambulatory care groups in the primary care of a European national health care system: does it work? Med Care 1999 37 3 238 248 10.1097/00005650-199903000-00004 10098568

[B24] Johnell K Merlo J Lynch J Blennow G Neighbourhood social participation and women's use of anxiolytic-hypnotic drugs: a multilevel analysis J Epidemiol Community Health 2004 58 1 59 64 10.1136/jech.58.1.59 14684728 PMC1757021

[B25] Johnson W Kyvik KO Mortensen EL Skytthe A Batty GD Deary IJ Education reduces the effects of genetic susceptibilities to poor physical health Int J Epidemiol 2010 39 2 406 414 10.1093/ije/dyp314 19861402

[B26] Braveman PA Cubbin C Egerter S Chideya S Marchi KS Metzler M Posner S Socioeconomic status in health research: one size does not fit all JAMA 2005 294 22 2879 2888 10.1001/jama.294.22.2879 16352796

[B27] Statistics Sweden, http://www.scb.se

[B28] Mwalili SM Lesaffre E Declerck D The zero-inflated negative binomial regression model with correction for misclassification: an example in caries research Stat Methods Med Res 2008 17 2 123 139 17698937 10.1177/0962280206071840

[B29] Anthony M Lee KY Bertram CT Abarca J Rehfeld RA Malone DC Freeman M Woosley RL Gender and age differences in medications dispensed from a national chain drugstore J Womens Health (Larchmt) 2008 17 5 735 743 10.1089/jwh.2007.0731 18537477

[B30] Schappert SM Burt CW Ambulatory care visits to physician offices, hospital outpatient departments, and emergency departments: United States, 2001–02 Vital Health Stat 13 2006 (159 1 66 159 16471269

[B31] Galdas PM Cheater F Marshall P Men and health help-seeking behaviour: literature review J Adv Nurs 2005 49 6 616 623 10.1111/j.1365-2648.2004.03331.x 15737222

[B32] Safran DG Rogers WH Tarlov AR McHorney CA Ware JE Gender differences in medical treatment: the case of physician-prescribed activity restrictions Soc Sci Med 1997 45 5 711 722 10.1016/S0277-9536(96)00405-4 9226794

[B33] Carr-Hill RA Rice N Roland M Socioeconomic determinants of rates of consultation in general practice based on fourth national morbidity survey of general practices BMJ 1996 312 7037 1008 1012 10.1136/bmj.312.7037.1008 8616346 PMC2350840

[B34] Adler NE Newman K Socioeconomic disparities in health: pathways and policies Health Aff (Millwood) 2002 21 2 60 76 10.1377/hlthaff.21.2.60 11900187

[B35] Carlsson L Strender LE Fridh G Nilsson GH Clinical categories of patients and encounter rates in primary health care - a three-year study in defined populations BMC Public Health 2006 6 35 10.1186/1471-2458-6-35 16483353 PMC1431523

[B36] Hjerpe P Merlo J Ohlsson H Bengtsson Bostrom K Lindblad U Validity of registration of ICD codes and prescriptions in a research database in Swedish primary care: a cross-sectional study in Skaraborg primary care database BMC Med Inform Decis Mak 2010 10 23 10.1186/1472-6947-10-23 20416069 PMC2864192

[B37] Medical Products Agency, http://www.lakemedelsverket.se/upload/nyheter/2012/Regeringsuppdrag%20olaga%20läke medel%202011-12-30.pdf

